# Assessment of hypercoagulability using thromboelastography predicts advanced status in renal cell carcinoma

**DOI:** 10.1002/jcla.23017

**Published:** 2019-08-22

**Authors:** Xun Wang, An Shi, Jiwei Huang, Yonghui Chen, Wei Xue, Jin Zhang

**Affiliations:** ^1^ Department of Urology, Renji Hospital Shanghai Jiaotong University School of Medicine Shanghai China

**Keywords:** advanced status, conventional coagulation tests, hypercoagulablity, renal cell carcinoma, thromboelastography

## Abstract

**Background:**

Thromboelastography (TEG) has been established as a sensitive method to assess the whole coagulation process. The aim of the study was to evaluate the diagnosis significance of TEG on hypercoagulability in patients suffering renal mass.

**Methods:**

A total of 478 patients were diagnosed with renal tumor by histolopathologic examination and were assigned to three groups. Group A: 79 patients with benign renal tumor; Group B: 317 patients with renal cell carcinoma (RCC, Fuhrman grades I and II); Group C: 82 patients with high‐risk RCC (Fuhrman grades III and IV). Subgroup analysis was performed in malignant renal tumor patients according to the TMN classification. The clinical data, whole blood TEG, and conventional coagulation tests were reviewed.

**Results:**

There was no statistically significant difference between subgroups in respect to conventional coagulation tests. Hypercoagulablity was marked in Group C according to the TEG parameters. The elevated platelets and fibrinogen is linked with hypercoagulability in renal tumor. The positive correlation was between fibrinogen and MA value (*r* = .663, *P* < .05). The pathologic tumor stages were also associated with the TEG parameters.

**Conclusion:**

Patients suffering advanced RCC are hypercoagulable which can be identified by TEG. MA value could be potential diagnosis indicators for detecting high‐grade RCC.

**Abbreviations**
APTT: activated partial thromboplastin time
NLR: Neutrophil‐to‐lymphocyte ratio
PT: prothrombin time
RCC: renal cell carcinoma
TEG: thromboelastography
TT: thrombin time
VTE: venous thromboembolism event

## 1 INTRODUCTION

It was once widely believed that hypercoagulablity was associated with malignancy.^1^ Hypercoagulability has been implicated in the pathogenic occurrence of venous thromboembolism events (VTE), which are the most common and serious non‐surgical complications after urological cancer surgery.^2^ The tumor cells may activate the coagulation process by releasing procoagulants, tissue factors, fibrinolytic proteins, or by invasion of the vessel wall, playing an important role in developing VTE in cancer patients.^3^ The incidence of VTE was reported to be from 0.14% to 1.67% in renal tumor patients.^4^, ^5^ A conventional coagulation test, which includes activated partial thromboplastin time (APTT) and prothrombin time (PT), is usually used to evaluate the coagulation status of patients. Several limitations were considered in these tests, including that all these tests are unable to reflect the overview of all aspects of the coagulation process.

Thromboelastography (TEG) was first introduced by Trousseau, and it is a type of graph that depicts the process from the beginning of clot formation to fibrinolysis.^6^ It has been established as a sensitive method to assess the entire coagulation process including clotting, platelet activation, kinetics of clot formation, and fibrinolysis. TEG has been widely used in guide transfusion, helping to evaluate the hemostasis function in patients with trauma and solid tumors.

The data concerning the clinical use of TEG in patients with renal tumor are rare. Therefore, we retrospectively reviewed renal mass patients who accepted operation in our clinical center and evaluated the coagulation status in those patients with solid renal mass for the first time using TEG data and conventional coagulation tests. The TEG data were also compared with routine coagulation analysis in order to determine the correlation between the two tests.

## 2 PATIENTS AND METHODS

We reviewed the records of patients who had undergone nephrectomy from July 2015 to February 2017 at RenJi Hospital affiliated with Shanghai Jiaotong University School of Medicine. All the mass‐bearing kidneys were detected by imaging and subsequently surgically removed within our institution. Patients with only complete pathological documentation and clinical data were included in this study. Patients taking anticoagulants and suffering preexisting hematological or coagulation disorder were excluded from this study. The clinical data including patient age, tumor site, stage (TNM classification), grade (Fuhrman classification), and histological subtype (angioleiomyolipoma, clear cell, papillary, etc) were recorded. TNM classification is based on the American Joint Committee on Cancer (AJCC 2010). In our study, tumors with Fuhrman grades I and II were classified as low grade. Grades III and IV were considered high grade. Patients were categorized into three subgroups by pathological results.

### 2.1 Laboratory assays

All blood tests were implemented within 24 hours before operation. The conventional coagulation testing, including APTT, PT, thrombin time (TT), and fibrinogen (FIB), was performed on the patients. Those parameters were determined by CA1500^®^（Sysmex co.,ltd. Japan）. All the reagents were manufactured by Siemens co.,ltd.: APTT（Actin, 0.025 mol/L CaCl_2_）, PT(Thromborel S), TT（Test Thrombin Reagent）, FIB(Thrombin Reagent 100NIH).Similarly, a complete blood cell analysis was also conducted, where attention was given to the platelet count and neutrolphil‐to‐lymphocyte ratio (NLR). Conventional TEG analysis was performed with the TEG 5000® (Haemoscope co.,ltd.). Five TEG parameters were measured as a presentation of coagulation status: R (reaction time, normal range: 3‐8 minutes); K time (normal range: 1‐3 minutes); α (alpha angle, normal range: 55° to 78°); MA (maximum amplitude, normal range: 51‐69 mm), and CI (coagulation index, normal range: −3 to 3).

### 2.2 Statistical analysis

Statistical analysis was performed using the Statistical Package for Social Sciences, version 22.0 (IBM Corp.). Continuous variables were described as means and standard deviation or as median value plus interquartile range (according to distribution). Continuous variables were analyzed using a one‐way analysis of variance (ANOVA) and the Kruskal‐Wallis test. The pairwise comparison *P* value resulted from a Wilcoxon rank‐sum test. The correlation analysis of TEG parameters and coagulation test were analyzed using Pearson's and Spearman correlation coefficient. All statistical tests were two sided, and the statistical significance was set at < 0.05.

## 3 RESULTS

A total of 478 patients were grouped as follows: Group A: 79 patients with benign renal tumor; Group B: 317 patients with renal cell carcinoma (RCC) (Fuhrman grades I or II); and Group C: 82 patients with high‐risk RCC (Fuhrman grades III and IV).

Demographic and pathological data including tumor stage are presented in Table 1. Patients in Group C had significantly higher levels of platelet count, NLR, and fibrinogen compared with the other two groups (*P* < .05). However, it should be noted that there was no difference among any other conventional coagulation test markers within these three groups (Table 2). When comparing Group C with Groups A and B, the analysis of TEG parameters showed a significant reduction of the R time and K time. Significant increases in MA, α angle, and CI were also found between Group C and the other groups, indicating that hypercoagulability was most evident in Group C (Table 3). In terms of TEG parameters, no difference was observed between Group A and B. The positive correlation was found between fibrinogen and MA value (*r* = .663, *P* < .05). There was also a weak correlation between platelet count and MA value (*r* = .423, *P* < .05). However, PT and TT failed to significantly correlate with any parameter of TEG. APTT weakly correlated with R time (*r* = .258, *P* < .05). As an inflammatory marker, NLR also presented the correlation with K time, alpha angle, and MA value. All results are summarized in Table 4.

**Table 1:** Baseline demographics of patients with renal benign and malignant mass

Characteristic	Group A	Group B	Group C	*P* value	A vs C	A vs B	B vs C
Number	79	317	82				
Female (n,%)	47, 59.4%	175, 55.2%	23, 28%	0.000	*	ns	*
Age (y)	54.1 ± 12.4	56.6 ± 11.9	61.7 ± 9.7	0.000	*	ns	*
Histopathological tissue type							
Angioleiomyolipoma	58	/	/				
Oncocytoma	4	/	/				
Leiomyoma	3	/	/				
Cyst	14	/	/				
Clear cell carcinoma	/	287	74				
Papillary cell carcinoma	/	12	7				
Chromophobe cell tumor	/	15	0				
Other renal cell carcinoma	/	3	1				

Note

ns means non‐significant.

* P<.05

**Table 2:** Conventional coagulation tests and neutrophil lymphocyte ratio for three group patients

	Group A	Group B	Group C	*P* value	A vs C	A vs B	B vs C
NLR	2.34 (1.72,3.13)	2.39 (1.83,3.29)	3.39 (2.56,4.54)	0.000	*	ns	*
PLT(x10^9^/l)	231.79 ± 59.32	213.57 ± 59.61	262.22 ± 92.47	0.000	*	ns	*
PT (s)	14.9 (13.6,17.7)	14.8 (13.7,17.1)	15.3 (13.3,17.6)	0.950			
APTT (s)	32.1 (28.7,34.9)	31.6 (28.8,33.4)	31.8 (28.8,36.2)	0.241			
TT (s)	10.5 (10.1,11.0)	10.4 (10.0,10.9)	10.6 (10.3,11.3)	0.626			
FIB (mg/dL)	2.86 ± 0.71	2.93 ± 1.03	4.23 ± 1.46	0.000	*		*

Note

ns means non‐significant.

* P<.05

**Table 3:** Major TEG parameters in the individual patient groups A, B, and C

	Group A	Group B	Group C	*P* value	A vs C	A vs B	B vs C
R time(s)	7.09 ± 1.08	6.91 ± 1.49	6.25 ± 1.44	0.001	*	ns	*
K time(s)	1.9 (1.4, 2.2)	1.8 (1.6, 2.2)	1.4 (1.0, 1.8)	0.000	*	ns	*
Alpha (degree)	69.08 ± 5.07	68.89 ± 5.18	73.27 ± 5.57	0.000	*	ns	*
MA (mm)	61.5 (59.0,64.9)	62.0 (57.5,65.6)	69.1 (65.8,76.2)	0.000		ns	*
CI	−0.5 (−1.4,0.9)	−0.1 (−1.5, 1,1)	1.7 (0.2,3.6)	0.000	*	ns	*

Note

ns means non‐significant.

* P<.05

**Table 4:** Correlation between TEG coagulation parameters and other laboratory data

		R	K	alfa	MA
PLT	Correlation	−0.136^a^	−0.405^b^	0.397^a^	0.423^b^
	Sig.(2‐tailed)	0.010	0.000	0.000	0.000
FIB	Correlation	−0.058^a^	−0.436^b^	0.450^a^	0.663^b^
	Sig.(2‐tailed)	0.275	0.000	0.000	0.000
PT	Correlation	0.012^b^	−0.024^b^	0.017^b^	−0.032^b^
	Sig.(2‐tailed)	0.815	0.654	0.750	0.542
TT	Correlation	0.067^b^	0.011^b^	0.020^b^	0.012^b^
	Sig.(2‐tailed)	0.207	0.962	0.742	0.847
APTT	Correlation	0.258^b^	0.098^b^	−0.097^b^	0.061^b^
	Sig.(2‐tailed)	0.000	0.065	0.066	0.249
NLR	Correlation	−0.074^b^	−0.134^b^	0.134^b^	0.219^b^
	Sig.(2‐tailed)	0.191	0.011	0.011	0.000

a Pearson's correlation coefficient

b Spearman correlation coefficient

During the postoperative period, VTE was identified in 3 people in group B and group C, consisting of 2 PE events and 1 DVT event.

To further analyze the hypercoagulability status of patients with RCC, the patients were additionally categorized into three subgroups according to the TMN classification: Group 1 (286 patients with pT1 tumor), Group B (74 patients with pT2 tumor), and Group 3 (patients with ≥ pT3 tumor) (Table 5). These three subgroups were also compared with the previously mentioned laboratory parameters. The result showed that the NLR, platelet count, and FIB level were all highest in Group 3. With respect to Groups 1 and 2, the significant decreased value in the R and K times, and the increase in MA, α angle, and CI were found in Group 3 (*P* < .05).

**Table 5:** The neutrophil lymphocyte ratio, conventional coagulation tests, and major TEG parameters in the individual patient groups 1,2, and 3

	Group 1	Group 2	Group 3	*P* value	1 vs 3	1 vs 2	2 vs 3
Number	286	74	39				
NLR	2.37 (1.88,3.29)	2.98 (1.64,3.79)	3.56 (2.47,4.80)	0.006	*	*	*
PLT (×10^9^/l)	211.97 ± 55.66	261.46 ± 103.36	267.38 ± 70.1	0.000	ns	*	*
PT (s)	15.0 (14.0,17.3)	15.0(13.4,18.1)	16.4(13.8,17.7)	0.637			
APTT (s)	31.0 (28.1,33.3)	31.6 (28.8,33.4)	31.6 (28.0,34.9)	0.103			
TT (s)	10.5(10.0,11.0)	10.8(10.0,11.7)	10.7(10.1,11.9)	0.265			
FIB (mg/dL)	2.94 ± 1.97	3.77 ± 1.44	4.22 ± 1.17	0.001	*	*	*
R time(s)	6.92 ± 1.61	6.69 ± 1.55	6.24 ± 1.03	0.129			
K time(s)	1.8 (1.5, 2.2)	1.7 (1.1, 2.0)	1.5 (1.15, 1.65)	0.000	ns	*	*
Alpha (degree)	68.65 ± 5.31	72.04 ± 6.31	73.89 ± 4.46	0.000	ns	*	*
MA (mm)	61.6 (57.5,65.2)	66.7 (60.7,71.1)	71.5 (65.9,74.0)	0.000	*	*	*
CI	−0.2 (−1.5,1.0)	0.8 (−0.6, 2.4)	1.7 (0.7,3.0)	0.000	*	ns	

Note

ns means non‐significant.

* P < .05

## 4 DISCUSSION

The results demonstrate hypercoagulable status finding in patients with high‐risk and high‐grade renal cancer (Fuhrman grades III and IV and ≥ pT2 tumor). The function of angiogenesis and coagulation is activated with tumor progression. Wang et al found that the local thrombin and plasmin information were closely related to tumor progression and metastases in patients with pancreatic cancer.^7^ This study also determined that the tumor cells could stimulate endothelial cells to release tissue factor and induce the secretion of fibrinolytic enzymes.^8^ The level of FIB, inflammatory factors (IL‐6), and D‐dimer were found in higher levels for those patients with metastasis. FIB was an important parameter showing the changes of clotting function and angiogenesis.^9^ Another study presented that the levels of FIB in patients with RCC were remarkably higher than those with benign renal tumors, where no significant differences were shown between the PT and APTT results.^10^ Similarly, our study also revealed the same results. By standard coagulation tests, we find it hard to detect the status of hypercoagulation in those patients with renal tumors. An elevation of the FIB was only found in groups with Fuhrman grades III and IV and ≥ pT2 stage. With the present investigation, the platelet counts were also found to be elevated in Group C and Group 3. Thus, we identified the value of platelets and fibrinogen in renal cancer‐induced hypercoagulability.

In other studies, it has been validated that TEG is a sensitive test to identify and evaluate the hypercoagulability status in patients with various types of cancer.^11^ Several studies have proved that the TEG parameters had strong ability in evaluating hypercoagulable status in patients with recurrent and advanced prostate cancer. This test was promising to be a novel tool for risk assessment of VTE in those patients.^12^, ^13^, ^14^ TEG is able to measure both the quantity and quality of clotting, providing the complete description of coagulation. Conventional coagulation tests only examine isolated parts of the coagulation cascade, thereby failing to contribute any important information regarding the clot and exhibiting limitations in the clinical evaluation of coagulation status.^15^ The clinical use of TEG to reveal the hypercoagulability status has been reported in patients undergoing general abdominal surgery,^16^ end‐stage renal failure,^17^ or cirrhosis.^18^ However, there are a few studies that analyze the TEG parameters in patients with RCC. Thus, our study was organized to compare the TEG results with routine coagulation tests in patients with renal tumor, specifically taking into consideration the coagulation status in those patients. With the present investigation, the group of patients with high‐risk RCC (Fuhrman grades III and IV) and high‐stage RCC (≥pT2 stage) had shorter R and K times, as well as increased α angle and MA and CI values, indicating increased thrombogenicity in those patients. Hypercoagulability in these patients may be induced by the presence of an accelerated clot formation and platelet function, as evidenced by the elevated α angle and MA value. It was reported that hyperfibrinogenemia and thrombocytosis—which are considered as two important prognostic factors—existed in patients with renal cell carcinoma.^10^, ^19^ Also, our results presented that K time, MA value, and α angle were significantly correlated with FIB. The positive correlation was found between fibrinogen and MA value (*r* = .663, *P* < .05), presenting the similarities with former studies focused on patients with localized prostate cancer.^14^ There was also a weak positive correlation between platelet count and MA value, suggesting that the MA value could both reflect the changes in platelet count and the state of platelet quality and function. TEG can provide additional clinical information with respect to platelet count.^20^ McCrath identified that the postoperative TEG MA levels showed the ability to predict the occurrence of postoperative thrombotic complication.^21^ It appears to be that the MA value can be identified as a signature parameter to identify the abnormal hemodynamics change in RCC patients.

Since it is widely believed that inflammatory reactions can induce cell proliferation to promote the occurrence of various tumors, as well as increase angiogenesis and tumor metastasis, NLR is often used for the evaluation of prognosis of multiple cancers. NLR was shown to also have the prognostic utility in both localized and metastatic renal cell carcinoma.^22^, ^23^ Since the process of thrombosis is relevant with inflammatory response, this study included NLR as an observational indicator. In our study, activated inflammation evaluated by NLR was also highly significant for patients categorized as Group C and Groups 2 and 3. In the correlation analysis, it was found that K time, MA, and α angle were significantly related with NLR, perhaps suggesting that the inflammation response may also play an important role in the coagulation reaction. The cytokines and chemokines released during inflammation process downregulate the expression of thrombomodulin and decrease the antithrombotic ability of endothelial cell, increasing the risk of thrombosis. Many inflammatory factors such as IL‐6, IL‐8, and MCP‐1 were found to be increased in vivo of VTE patients.^24^

The application of a single laboratory examination item usually has specific limitations and does not fully reflect the entire disease status. Accordingly, we believe the thromboelastogram and conventional coagulation test can complement each other in terms of clinical use. A comprehensive analysis of the entire coagulation process and systematic analysis will provide additional guidance in the diagnosis and treatment of coagulation abnormalities in patients with renal tumors.

The limitations of the present study include the retrospective analysis of RCC cases and heterogeneity of the series. The results may only reflect a single‐center experience. The main goal of our study was to present the coagulation status in patients with RCC. As such, we endeavored to provide evidence that TEG could be applied to evaluate the RCC‐induced hypercoagulability status.

## 5 CONCLUSION

Patients with advanced renal tumors are hypercoagulable. The TEG parameters are effective hemodynamic markers in evaluating this abnormal hemodynamic status in RCC patients, which is superior to the conventional coagulation tests. MA value appeared to be a signature parameter to identify the abnormal hemodynamics change in RCC patients. It should be noted that an increased MA has a close link with those prognostic markers in RCC. On the basis of our funding, further study may emphasize the use of TEG in identifying those with risk for venous thrombosis events (VTE) during disease progression.

## CONFLICT OF INTERESTS

All authors read and approved the final manuscript. The authors declare that they have no competing interests.

## AUTHORS' CONTRIBUTIONS

X Wang: Manuscript writing, Data analysis, Data collection. A Shi: Data collection, Data analysis. JW Huang: management. YH Chen: management. W Xue: Polishing manuscript. J Zhang: project development, management.

## ETHICS APPROVAL AND CONSENT TO PARTICIPATE

Our study was approved by the institutional review board of Renji Hospital affiliated to Shanghai Jiaotong University School of Medicine, and the requirement for informed consent was waived.

## CONSENT PUBLICATION

Not applicable.

## Data Availability

The datasets used and analyzed during the current study available from the corresponding author on reasonable request.
